# Urbanization-Induced Shifts in Microbial Functional Genes of Wetland Nitrogen Cycling Promote Nitrous Oxide (N_2_O) Emissions

**DOI:** 10.3390/microorganisms14030640

**Published:** 2026-03-12

**Authors:** Xinyu Yi, Yuwen Lin, Yinghe Peng, Yan Liu, Chen Ning, Junjie Lei, Ling Wang, Chan Chen, Linshi Wu, Juyang Liao

**Affiliations:** 1 Hunan Botanical Garden, Hunan Changsha-Zhuzhou-Xiangtan City Cluster Ecosystem Observation and Research Station, Changsha 410116, China; yixinyu1108@163.com (X.Y.); liuyan6287@163.com (Y.L.); w19968@163.com (L.W.); chenchan0829@163.com (C.C.); wulinshi1990@163.com (L.W.); 2 College of Agriculture, Nanjing Agricultural University, Nanjing 210095, China; linyuwen0929@163.com; 3 Forestry Department of Hunan Province, The Forestry Affairs Center of Hunan Province, Changsha 410004, China; pyinghe@163.com; 4 National Engineering Laboratory of Applied Technology for Forestry and Ecology in Southern China, Central South University of Forestry and Technology, Changsha 410004, China; ningchen0059@163.com (C.N.); lailxy1314@126.com (J.L.)

**Keywords:** urban wetlands, nitrous oxide, nitrogen cycle, functional genes

## Abstract

Urban wetlands are assumed to contribute to nitrous oxide (N_2_O) emissions; however, the microbial mechanisms underlying enhanced N_2_O fluxes in urban wetlands and differences in microbial responses between aquatic and soil compartments have not been clearly identified. Here, we characterized the nitrogen (N) cycling microbial communities and their functional metabolic pathways in urban and rural wetlands using metagenomics and N_2_O flux measurements. Results showed that urbanization drove a 6~8-fold increase in N_2_O fluxes from urban wetlands compared to rural wetlands. Structural equation modeling (SEM) confirmed that urbanization intensity was a primary driver (standardized coefficients: 0.72 for soil and 0.92 for water). In wetland water, N_2_O emissions were negatively correlated with inorganic nutrient concentrations (coefficient = −0.62). Aquatic microbial communities exhibited substantial taxonomic shifts but preserved network connectivity, indicating adaptive strategies for surviving urban perturbations at the cost of reduced functional redundancy. In wetland soil, microbial communities maintained stability under urbanization, which was attributed to environmental buffering from heterogeneous microenvironments. Soil N_2_O emissions were positively linked to microbial alpha diversity (coefficient = 0.79). Furthermore, urban wetlands enriched genes mediating nitrification and denitrification while depleting genes associated with N fixation and organic N metabolism. This functional shift reflects microbial specialization in processing elevated reactive N (Nr) inputs from urban sources, trapping urban wetlands in an “N loss loop” that reinforces high N_2_O fluxes. This study elucidates the microbial mechanisms governing wetland N_2_O emissions under urbanization, thereby enhancing understanding of microbially mediated N cycling in the urban wetland ecosystem.

## 1. Introduction

Wetlands, which store approximately 30% of the global terrestrial soil nitrogen (N), are important terrestrial N pools and play a pivotal role in regulating the global N cycle [1,2]. Urbanization collectively causes disturbances and leads to dramatic changes in ecosystem functions [3]. Over the past decades, intensified human activities, such as increased industrial and domestic wastewater discharge, excessive application of N-based fertilizers, and fossil fuel combustion, have significantly increased anthropogenic emissions of reactive N and N pollution in wetlands [4,5], disrupting natural N-cycling processes and altering the structure and function of wetland ecosystems [6,7]. Urban wetlands, characterized by N pollution, elevated temperature, and land use change, have been identified to significantly alter N transformation processes and further accelerate N_2_O emissions [8]; however, their role in the global N cycling remains unclear.

Microbe-driven N removal pathways play a crucial role in maintaining global N balance, and microbial processes contribute over 60% of natural N_2_O emissions [9,10]. Microbial N-transforming processes are influenced by various environmental parameters [11,12,13]. For example, the abundance and structure of N cycle genes are strongly regulated by soil pH [14,15]; elevated temperature has been found to increase the abundance of *nirK* and *nosZ* genes [16]; and long-term N fertilizer application has increased the abundance of microorganisms involved in N-transforming processes but decreased that of N-fixing assemblages [17]. Urbanization significantly alters N cycle processes, which are closely linked to the regulation of microbial functional genes [18,19]. For instance, the *nirK* and *nirS* genes are involved in NO_3_^−^ consumption and N_2_O production, while *nosZ* regulates the conversion of N_2_O to N_2_ during denitrification [20]. Recent studies have characterized the taxonomic composition and distribution of bacterial and archaeal communities under urbanization [21,22,23,24]. However, the effects of urbanization intensity on wetland N_2_O emissions, particularly on the composition of N_2_O-associated functional communities, remain poorly understood. Furthermore, most studies have focused on individual gene functions (e.g., *nirK*/*nirS*-mediated N_2_O production) and a single wetland matrix (e.g., soil or sediment), but few have systematically explored how the abundance and interactions of these functional genes collectively respond to urbanization intensity.

In our previous work, we characterized an urban–rural gradient by impervious surface area (ISA) across 20 wetlands in Hunan Province, documenting changes in microbial taxonomic composition using 16S rRNA sequencing [22]. Building on these findings, we selected six of these wetlands to quantify in situ soil and water N_2_O fluxes and collect soil and water samples. We employed an integrated approach combining marker gene analysis and metagenomics to comprehensively explore microbial functional potentials and networks, as well as their associations with N_2_O fluxes in urban and rural wetlands. We aimed to investigate how microbial communities and their N functional genes respond to urbanization, whether wetland N_2_O emissions differ between urban and rural wetlands, and how functional genes and associated environmental factors contribute to N_2_O emissions. We hypothesized that (1) N_2_O fluxes differ significantly between urban and rural wetlands and are linked to contrasting environmental conditions, (2) compared to rural wetlands, urbanization significantly alters the composition of microbial taxa involved in the N cycle and increases the functional potential of N-cycling genes in wetlands, and (3) the diversity and abundance of N-cycling functional genes differ significantly between urban and rural wetlands, with these differences varying between soil and water compartments.

## 2. Materials and Methods

### 2.1. Study Area Selection and Sample Collection

**Figure 1:**
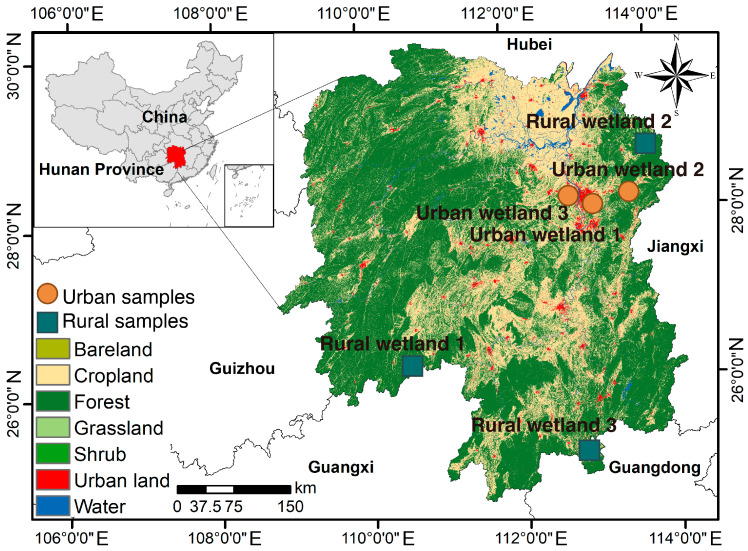
Map of rural and urban wetland sampling sites across Hunan Province, China. Rural wetlands 1, 2, and 3: sampling sites 1, 2, and 3 in rural wetlands. Urban wetlands 1, 2, and 3: sampling sites 1, 2, and 3 in urban wetlands, respectively.

The study was conducted in Hunan Province, China, which is located in the middle reaches of the Yangtze River (243°8′ N to 30°08′ N, 108°47′ E to 114°15′ E) (Figure 1, Table S1). This region has a subtropical humid monsoon climate, characterized by a mean annual temperature of 16.5 °C and an annual precipitation of 1448 mm. The wetland soils are classified as Luvisols (IUSS Working Group WRB, 2014) with a silty clay loam texture. Field sampling was performed in April 2023, covering 36 samples (18 soil and 18 water samples) from six wetland sites along the urban–rural gradient, with three replicates per site. Urban wetland sites (UR1, UR2, and UR3) were situated in a typical urbanizing area, which has experienced intense anthropogenic disturbances over the past 10 years, including domestic sewage discharge, construction activities, and agricultural runoff. Rural wetland sites (RU1, RU2, and RU3) were located in a natural wetland with minimal human interference, serving as reference sites.

Soil samples were collected from the 0~20 cm depth. All samples were thoroughly mixed and immediately stored in sealed bags for transportation to the laboratory. A 10 g soil sample of each sample was immediately frozen in liquid nitrogen for 2 h and then stored at −80 °C for subsequent metagenomic analysis. The remaining soil samples were passed through a 2 mm sieve to remove plant residues and gravel and then divided into two fractions: one fraction was air-dried at room temperature (25 °C) for physicochemical property analysis, and the other was stored at 4 °C for the determination of soil microbial biomass carbon (MBC) and nitrogen (MBN).

For water sample collection, 1000 mL of surface water (0~20 cm depth) was sampled using a sterile sampling bottle at each site. Prior to sampling, the bottle was rinsed three times with in situ water to minimize contamination. The water samples were thoroughly mixed to homogenize the bacterioplankton community, then filtered through a 250 μm nylon mesh to remove large debris, followed by filtration through 0.22 μm polycarbonate membrane filters (47 mm diameter, Whatman, Maidstone, UK). The filters containing microbial biomass were stored at −80 °C until DNA extraction.

All sampling plots were established in bare areas to eliminate the confounding effects of plant-related N_2_O production/consumption processes. The selected wetlands were matched for key environmental attributes: hydrological regime (shallow marsh wetlands with water depth ranging from 0.5 to 1.2 m), soil parent material, vegetation coverage (<5%, dominated by herbaceous plants), and climatic conditions.

### 2.2. Determination of Soil and Water Physicochemical Properties

In situ measurements of water temperature (WT), pH, dissolved oxygen (DO), oxidation-reduction potential (ORP), electrical conductivity (EC), and total dissolved solids (TDS) were conducted using a multi-parameter analyzer (YSI ProPlus, Yellow Springs, OH, USA). For soil pH determination, a soil-water suspension (1:2.5 *w*/*v*) was prepared and equilibrated for 60 min, then measured using a pH meter (INESA instruments Inc., Shanghai, China).

Soil MBC and MBN were determined by the chloroform fumigation–extraction method [25]. Soil organic carbon (SOC) was analyzed using the wet oxidation method with potassium dichromate–sulfuric acid. Total nitrogen (TN) and total phosphorus (TP) in soil and water samples were determined by alkaline potassium persulfate digestion followed by ultraviolet spectrophotometry (UV-2600, Shimadzu, Kyoto, Japan). Ammonium nitrogen (NH_4_^+^-N) and nitrate nitrogen (NO_3_^−^-N) were extracted with 1 M KCl (1:5 *w*/*v*) and measured using a continuous-flow automated analyzer. Contents of heavy metals (Zn, Pb, Cr, Cu, Hg, and As) in soil samples were determined by microwave-assisted digestion with a mixed acid system (HNO_3_-HCl-HF, 5:3:2, *v*/*v*/*v*) (CEM, Mars 6, Matthews, NC, USA), followed by detection using inductively coupled plasma mass spectrometry (ICP-MS, NexION 350X, PerkinElmer, Waltham, MA, USA) [26]. Water sulfate (SO_4_^2−^) was analyzed by ion chromatography (ICS-600, Thermo Fisher Scientific, Waltham, MA, USA) equipped with an IonPac AS11-HC column.

### 2.3. Greenhouse Gas Flux Measurement

N_2_O fluxes were quantified using an LI-7810 trace gas analyzer (LI-COR Biosciences, Lincoln, NE, USA) during the sampling period (April 2023). A total of 10 soil collars were pre-installed in the sampling plots 24 h prior to measurement to stabilize the soil microenvironment. The chamber had a total volume of 5245.06 cm^3^ (20 cm diameter). All gas flux measurements were synchronized with the soil sampling to ensure consistency of environmental conditions. For the N_2_O concentration change rate calculation, a 2-min moving window was applied to analyze the 5-min continuous measurement data, and linear regression was performed to fit the concentration dynamics (data with r^2^ < 0.8 were discarded as invalid). For water surface flux measurements, a foam-buoyed floating chamber (volume: 4244.10 cm^3^) was used, and the measurement area was ensured to be free of floating vegetation. Flux calculation was implemented via SoilFluxProTM software (version 5.2.0, LI-COR Biosciences).

### 2.4. DNA Extraction, Library Preparation, and Metagenomic Sequencing

Total genomic DNA was extracted from soil and filter samples using an E.Z.N.A.^®^ Soil DNA Kit (Omega Bio-tek, Norcross, GA, USA) following the manufacturer’s protocol. The concentration and purity of extracted DNA were quantified with TBS-380 and NanoDrop 2000 systems, respectively. DNA extract quality was checked on a 1% agarose gel.

For metagenomic library construction, 1 μg of high-quality DNA was sheared into fragments of approximately 400 bp using a Covaris M220 Focused-Ultrasonicator (Gene Company Limited, Shanghai, China). The fragmented DNA was subjected to end repair, A-tailing, and adapter ligation using the NEBNext Ultra DNA Library Prep Kit for Illumina (New England Biolabs, Ipswich, MA, USA). The ligated products were purified and amplified by PCR to generate the final libraries. Library quality was assessed using an Agilent 2100 Bioanalyzer (Agilent Technologies, Santa Clara, CA, USA) and quantitative real-time PCR (qPCR).

High-throughput sequencing was performed on an Illumina NovaSeq Xten platform (Illumina, San Diego, CA, USA) with paired-end 150 bp reads at Majorbio Bio-Pharm Technology Co., Ltd. (Shanghai, China) using NovaSeq/HiSeq X reagent kits according to the manufacturer’s instructions (www.illumina.com). After sequencing, raw reads were filtered to remove adapter sequences, low-quality reads (Q-score < 20), and reads containing more than 5% ambiguous bases. A total of 1,802,410,934 clean reads (average 50,066,970 reads per sample) were obtained (Table S6). The raw sequence data have been deposited in the NCBI Sequence Read Archive (SRA) database under the accession number SRPPRJNA1021146.

### 2.5. Data Processing and Statistical Analysis

All statistical analyses were performed using R software (version 3.6.0) with the following packages: *vegan*, *ggplot 2*, *lavaan*, and *igraph* [27]. Prior to analysis, all data were tested for normality using the Shapiro–Wilk test and homogeneity of variance using Levene’s test. Variables with different units were standardized using Z-score standardization to eliminate the influence of dimensionality.

Differences in environmental factors, microbial diversity indices, and N_2_O fluxes were analyzed using one-way analysis of variance (ANOVA) followed by least significant difference (LSD) multiple comparison tests (*p* ≤ 0.05). Nonmetric multidimensional scaling (NMDS) based on Bray–Curtis dissimilarity was used to visualize the differences in N-cycling functional gene communities between urban and rural wetlands, and analysis of similarity (ANOSIM) was performed to test the significance of these differences.

Spearman’s correlation analysis was used to explore the relationships between environmental factors and functional gene abundances. Redundancy analysis (RDA) was conducted to identify the key environmental drivers shaping the functional gene communities. Co-occurrence networks of N-cycling microbes were constructed based on Spearman’s correlation coefficients (|r| > 0.6 and *p* < 0.05) after Benjamini–Hochberg correction. Network topological parameters (node number, edge number, average degree, clustering coefficient, and modularity) were calculated using *igraph* [28], *Hmisc* [29], and *Matrix* [30], and the network was visualized using Gephi software 0.10.1 [31]. To assess the statistical significance of the observed network structure, we constructed null models based on the configuration model as randomized controls. For each observed network, 1000 random networks were generated, preserving the original number of nodes and the degree (number of connections per node) while randomly rewiring the edges. The modularity, average clustering coefficient, and average path length were computed for each random network using the Louvain algorithm. The observed values were then compared to the null model distribution, and statistical significance was evaluated by calculating Z-scores and empirical *p*-values. All analyses were performed in the R environment using the *igraph* package.

A structural equation model (SEM) was constructed using the lavaan package in R to quantify the direct and indirect effects of urbanization intensity, environmental factors (soil and water properties), and functional gene abundances on N_2_O fluxes. The initial model was developed based on theoretical hypotheses, prior related studies, and the actual ecological processes of N_2_O emission. Variable selection and latent constructs were determined as follows: urbanization intensity was selected as a key exogenous variable because it directly drives changes in wetland environmental conditions and microbial communities [22]; soil properties (TOC, TN, TP, MBC, MBN), water properties (DO, K, NO_3_^−^-N, NH_4_^+^-N, PO_4_^3−^, SO_4_^2−^), heavy metals (Pb, Cu, Cd, As), and microbial diversity indices (Shannon, Sobs) were grouped into latent constructs (collinearity was pre-tested, variance inflation factor < 5 to avoid model instability); functional gene abundances (including denitrification and nitrification genes) were included as endogenous variables, as they directly mediate microbial N-cycling processes related to N_2_O fluxes. A total of 36 samples were used for SEM construction. Although the number of sampling sites was limited, we minimized potential instability by reducing model complexity (avoiding excessive latent constructs and observed variables) and pre-testing variable collinearity. Model fitting was evaluated using multiple indices: root mean square error of approximation (RMSEA < 0.08), comparative fit index (CFI > 0.90), and standardized root mean square residual (SRMR < 0.05) [32]. Non-significant paths (*p* > 0.05) were removed iteratively to optimize the model fit.

## 3. Results

### 3.1. Effects of Urbanization on N_2_O Fluxes and Environmental Factors

**Figure 2:**
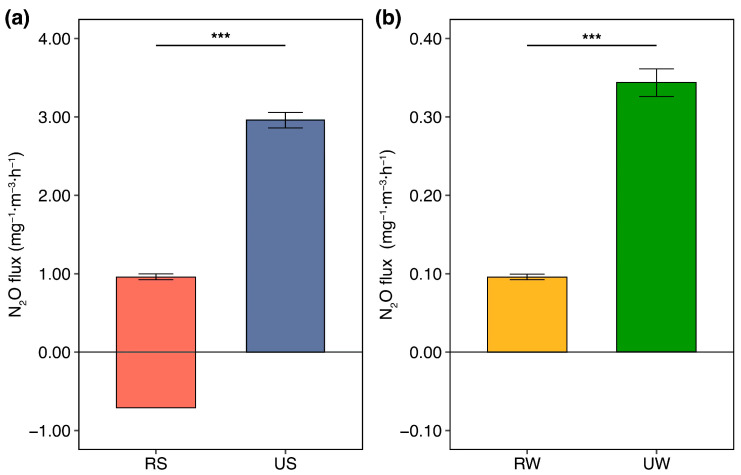
(**a**) N_2_O fluxes at the soil-atmosphere interface in wetlands under urbanization; (**b**) N_2_O fluxes at the water-atmosphere interface in wetlands under urbanization. RS: rural wetlands soil; US: urban wetlands soil; RW: rural wetlands water; UW: urban wetlands water. *** *p* < 0.001.

Urbanization drove a 6~8-fold increase in N_2_O fluxes from urban wetlands compared to rural wetlands. The average N_2_O fluxes at the soil-atmosphere interface were significantly higher in urban wetlands (1.338 mg^−1^·m^−3^·h^−1^) than in rural wetlands (0.21 mg^−1^·m^−3^·h^−1^) (Figure 2). At the water–atmosphere interface, the highest N_2_O fluxes (0.343 mg^−1^·m^−3^·h^−1^) were recorded at the urban wetland, while the lowest N_2_O fluxes (0.04 ± 0.002 mg^−1^·m^−3^·h^−1^) were observed in the rural wetland.

For soil properties, soil pH and SO_4_^2−^ content were significantly higher in urban wetlands than in rural wetlands (*p* < 0.05) (Table S5). In contrast, other soil properties, including TOC, MBC, MBN, NH_4_^+^-N, and NO_3_^−^-N contents, were significantly lower in urban wetlands (*p* < 0.05). Specifically, MBC and MBN in rural wetlands were approximately 3-fold higher than those in urban wetlands.

For water properties, urban wetlands exhibited significantly higher contents of heavy metals, nutrients, and available N than rural wetlands (*p* < 0.05) (Table S4). Additionally, the physical properties of water, DO, EC, and TDS, were also significantly higher in urban wetlands compared to their rural counterparts (*p* < 0.05).

### 3.2. Effect of Urbanization on N-Cycling Functional Community Structure and Diversity

**Figure 3:**
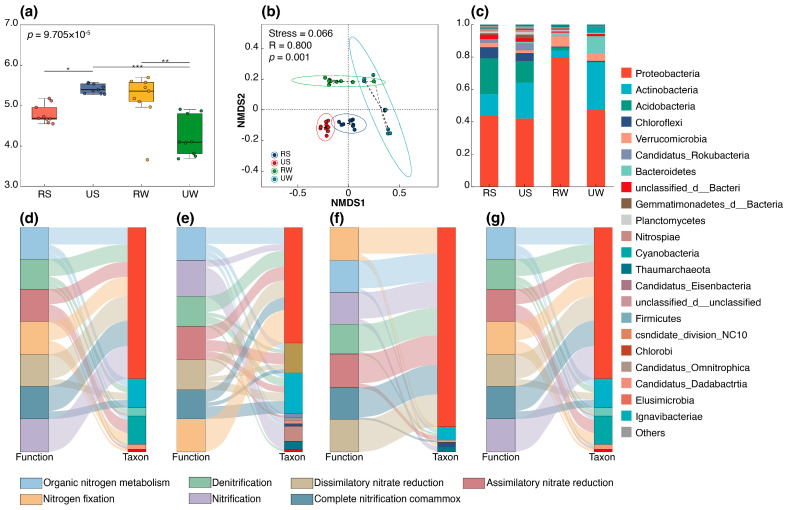
Microbial community characteristics and N cycling functional contributions in wetlands under urbanization. (**a**) Alpha diversity expressed by the Shannon index. (**b**) Non-metric multidimensional scaling (NMDS) analysis based on Bray–Curtis distances. (**c**) Relative abundances of the top 22 phyla. (**d**–**g**) Sankey diagram of phylum-level taxa contributions to N cycling in rural wetland soil samples, urban wetland soil samples, rural wetland water samples, and urban wetland water samples, respectively. * *p* < 0.05, ** *p* < 0.01, *** *p* < 0.001.

The Shannon index values of N-cycling genes were significantly higher in urban wetland soils than in rural samples (Figure 3a), whereas an opposing trend was observed in wetland water. NMDS indicated significant compositional differences in N-cycling gene communities between urban and rural water samples (Figure 3b, stress = 0.066), whereas such spatial differentiation was less pronounced in soil samples. Among the functional microbial communities in the wetlands, the five most abundant phyla were Proteobacteria, Actinobacteria, Acidobateria, Chloroflexi, and Verrucomicrobia. Proteobacteria emerged as the predominant contributor to the 23 annotated N-cycling genes, accounting for 41.94~80% of total gene abundances (Figure 3c). Its relative abundance remained stable across soil samples (Kruskal–Wallis test, *p* = 0.627), whereas a significant decline was observed in urban wetland water (*p* = 0.03). Furthermore, urbanization also increased the relative abundances of Actinobacteria, Bacteroidetes, and Cyanobacteria.

Proteobacteria were the main contributors to the considered functions, with their relative contributions in urban wetlands significantly decreased compared with those in rural wetlands (across seven functional categories, the values ranged from 0.44 to 0.39 in soil samples and from 0.68 to 0.536 in water samples for rural and urban sites, respectively (Table S9) (*p* < 0.05, Figure 3d–g). The taxa affiliated with Actinobacteria and Cyanobacteria exhibited higher contributions in urban water samples, accounting for 0.65 and 0.86, respectively (Figure 3f,g). Notably, the relative contributions of Cyanobacteria to organic N metabolism and anaerobic ammonium oxidation (ANRA) were significantly increased in urban wetland water compared to rural water samples, reaching 0.116 and 0.398, respectively.

**Figure 4:**
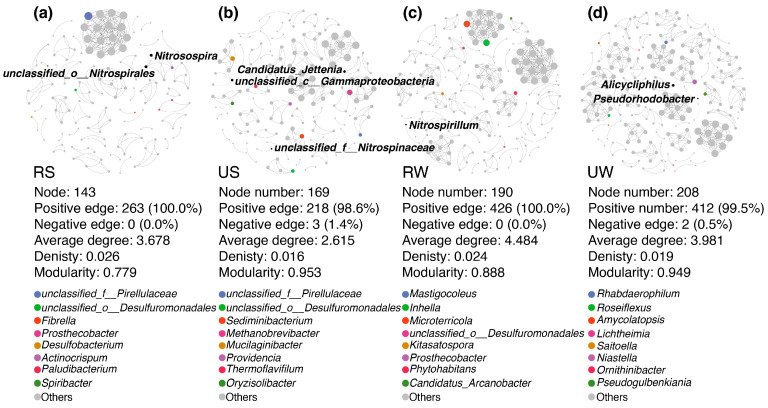
Network of microbial associations in rural and urban wetlands. (**a**–**d**) Interaction networks of the top 8 genera by relative abundance in (**a**) rural wetland soil samples, (**b**) urban wetland soil samples, (**c**) rural wetland water samples, and (**d**) urban wetland water samples, respectively. Node sizes indicate the degree of relative abundance. Edge colors indicate interspecific correlations: red edges represent negative correlations, and gray edges represent positive correlations. Edge thickness reflects correlation strength.

Co-occurrence networks were constructed to analyze dominant N_2_O-associated microbial genera and assess inter-taxon associations (Figure 4a–d). Despite variations in node and edge numbers between rural and urban wetlands, species interactions remained relatively stable. Null model analysis showed significant differences in modularity between urban and rural wetlands (Table 1). Significant modularity was observed in rural soil (Z = 2.188, *p* = 0.046) and water samples (Z = 2.221, *p* = 0.037). In contrast, no significant modularity was detected in urban soil (Z = 0.445, *p* = 0.253) or water samples (Z = 0.128, *p* = 0.428).

### 3.3. Effect of Urbanization on N-Cycling Functional Genes

**Figure 5:**
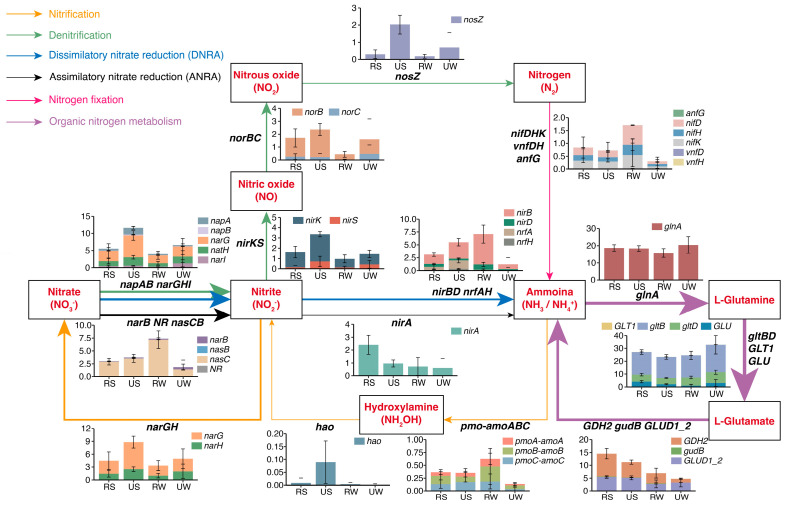
Relative abundance of associated functional genes in N metabolism pathways in wetlands. Functional genes in wetland samples were annotated using the Kyoto Encyclopedia of Genes and Genomes (KEGG) and visualized as bar plots, with values representing mean ± standard deviation, %. Arrow thickness and color represent relative abundance and different metabolic pathways, respectively.

No functional genes (*hzs* and *hdh*) associated with anammox were detected; thus, the anammox process was excluded from further analyses. The data revealed that genes involved in organic N metabolism dominated in wetland ecosystems, accounting for 54.9% of the total functional genes, with the *glnA* and *gltB* genes being notably abundant in all samples (Figure 5, Table S10). Functional genes involved in organic N metabolism (e.g., *GDH2*, *gltB*), N fixation (e.g., *nifD*), and ANRA (e.g., *nirA*, *nasC*) exhibited relatively lower abundances in urban wetlands compared with rural wetlands. Notably, urbanization was associated with marked increases in the potential of nitrification and denitrification pathways, and the N_2_O-associated genes (e.g., *narG*, *nirS*, *nirK*, *norB*, *norC*, and *hao*) were dominant in urban wetlands, representing the most abundant functional gene group in urban wetland soils. For example, the relative abundances of the *nirK* and *nirS* genes increased from 0.63% and 0.06% to 1% and 0.27%, respectively, in urban soil samples.

### 3.4. Drivers of N Cycling and Their Linkages

**Figure 6:**
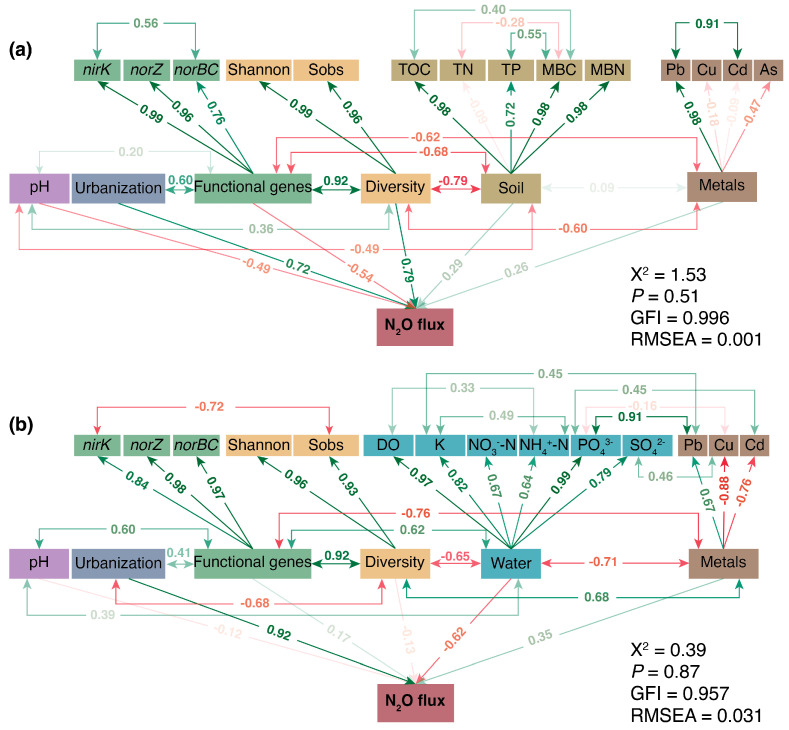
The structural equation model (SEM) identifies the direct relationship between N_2_O flux and biotic and abiotic factors in wetlands. (**a**) SEM results for fluxes in soil samples; (**b**) SEM results for fluxes in water samples. Green and red arrows indicate positive and negative effects, respectively (*p* < 0.05). The transparency of the arrows and numbers indicates the effect size of the relationships. The model’s fit was evaluated using standard goodness-of-fit metrics (χ^2^, P, GFI, and RMSEA). GFI, goodness-of-fit index; RMSEA, root mean square error of approximation.

SEM analysis confirmed that urbanization exerted stronger direct than indirect effects on N_2_O emissions, with standardized coefficients of 0.72 at the soil–atmosphere and 0.92 at the water–atmosphere interfaces (Figure 6a,b). At the water–atmosphere interfaces, urbanization showed a stronger direct association with N_2_O flux, which was strongly influenced by changes in inorganic nutrients (e.g., PO_4_^3−^, SO_4_^2−,^ and NO_3_^−^-N), with a standardized coefficient of −0.62. For the soil–atmosphere interfaces, changes in the soil environment observed in wetlands were linked to N_2_O fluxes, with this association related to shifts in microbial diversity indexes and the abundance of functional genes in N-cycling pathways.

## 4. Discussion

### 4.1. Microbial Mechanisms Underlying Increased N_2_O Flux in Urban Wetlands

Our results highlighted that urban wetlands emitted significantly more N_2_O from both soil and water than rural wetlands. This observation was strongly supported by SEM analysis, which quantified the direct impacts of urbanization intensity on N_2_O emissions; the standardized coefficients were 0.72 at the soil–atmosphere interface and 0.92 at the water–atmosphere interface. These results underscore that urbanization-driven land use changes act as primary drivers amplifying N_2_O release in urban wetlands. Notably, N_2_O emissions from urban wetland water are strongly regulated by changes in inorganic nutrients (PO_4_^3−^, SO_4_^2−^, and NO_3_^−^-N), with a negative coefficient of −0.62. This potentially reflects intensified nutrient loading from urban runoff that disrupts microbial N transformation pathways. For example, high concentrations of PO_4_^3−^ carried by urban surface runoff may alter the redox environment in the water, thereby inhibiting the respiratory metabolism of denitrifying bacteria. Previous studies have shown that excessive phosphorus input promotes the massive proliferation of phytoplankton, such as cyanobacteria, leading to drastic fluctuations in dissolved oxygen (DO) [33,34]. The denitrification process is extremely sensitive to DO levels; the instability of hypoxic conditions directly reduces the completeness of NO_3_^−^-N conversion to N_2_ by denitrifying bacteria, thereby increasing the release of N_2_O as an intermediate product [35].

Soil N_2_O emissions were positively linked to microbial alpha diversity, with a coefficient of 0.79, suggesting that urbanization-induced shifts in soil microbial communities may enhance N_2_O-producing processes. The significant differences in aquatic species composition between urban and rural wetlands, coupled with stable network relationships in both urban and rural wetlands, indicate that urbanization drives ecological restructuring without destabilizing overall community interactions, yet this restructuring favors N_2_O emission potential [36,37].

The reduction in functional genes related to N fixation and organic N metabolism in urban wetlands further highlights a shift in N-cycling strategies. Urban wetlands appear to prioritize inorganic N transformations (nitrification/denitrification) over N retention or organic matter processing. This shift may be associated with enhanced N_2_O release, as the prioritization of these inorganic transformations could favor incomplete denitrification or elevated potential for nitrification under nutrient-rich conditions. This functional gene shift, combined with the strong direct association of urbanization with N_2_O emissions, suggests that urban wetlands are trapped in an “N loss loop”, where increased nutrient inputs and altered microbial functions could reinforce high N_2_O fluxes [38,39]. Importantly, the stability of network relationships implies that even modified microbial communities retain potential functional redundancy, but their reorganized structure appears to prioritize processes potentially linked to greenhouse gas emissions [40]. These results highlight the need for targeted management strategies, such as reducing nutrient inputs to aquatic systems and preserving soil microbial diversity, to mitigate N_2_O emissions from urban wetlands while maintaining ecosystem functionality.

### 4.2. Differences in N-Cycling Microbial Functional Genes and Metabolic Pathways in Urban Wetlands

Urban wetlands exhibit distinctive microbial functional adaptations in N cycling, which are fundamentally shaped by urbanization-induced environmental filters [24]. The increased abundance of functional genes associated with denitrification and nitrification implies a potential enhancement in metabolic activity, which may be linked to elevated N_2_O production and emission. Furthermore, the higher abundance of these functional genes provides insights into how microbial communities respond to the N surplus characteristic of urban wetlands. This functional shift represents a form of microbial specialization for processing elevated reactive Nr inputs from anthropogenic sources, consistent with the selective pressures imposed by increased ammonium and nitrate availability. The enrichment of denitrification (e.g., *nirS*, *nirK*, *nosZ*) and nitrification (e.g., *amoA*, *hao*) genes aligns with the elevated N loading typical of urban wetlands. Urban wetlands receive substantial Nr inputs from stormwater runoff, sewage leakage, and atmospheric deposition, often resulting in high concentrations of ammonium (NH_4_^+^) and nitrate (NO_3_^−^) in sediments and porewater [41,42,43]. This surplus Nr exerts strong selective pressure favoring microbial taxa specialized in Nr transformation; nitrifiers (e.g., Nitrosomonas, Nitrobacter) thrive under elevated NH_4_^+^, while denitrifiers benefit from increased NO_3_^−^ as an electron acceptor in anaerobic microsites [44]. This adaptive response positions urban wetlands as potential hotspots for Nr transformation, although complex trade-offs involving greenhouse gas emissions merit further investigation. Concurrently, the reduced abundance of genes associated with N fixation (e.g., *nifH*) and organic N metabolism (e.g., *ureC*, *gdh*) indicates a reduced potential capacity for organic N acquisition pathways. Urban wetlands often experience shifts in organic matter composition; for example, terrestrial plant litter inputs decline due to landscape fragmentation, whereas labile organic carbon from anthropogenic sources (e.g., detergents, sewage) increases [43]. This alteration in carbon quality may reduce the competitive advantage of diazotrophs, which typically require substantial energy (ATP) for N_2_ fixation, a process that becomes less favorable when Nr is readily available [45]. Therefore, changes in N_2_ fixation-related processes may further affect the nutrient storage capacity of the wetland, which in turn influences wetland stability.

These functional gene dynamics collectively reshape N-cycling pathways in urban wetlands, shifting the ecosystem toward a more “reactive” N economy. A potentially enhanced nitrification-denitrification loop may strengthen the role of wetlands as Nr sinks and mitigate downstream eutrophication. However, this service depends on sufficient carbon availability to sustain denitrification [46]. The concurrent reduction in genes linked to N fixation and organic N metabolism may reduce the capacity to cope with Nr limitation in urban wetlands where N inputs decline. Furthermore, a decoupling between microbial processing of inorganic and organic N could alter microbial food webs, as shifts in N sources may exert cascading effects on primary producers and higher trophic levels dependent on microbial N transformation processes [47,48]. In the natural wetlands, N fixation typically plays a critical role in sustaining primary productivity under low Nr conditions, with organic N mineralization representing the dominant pathway of N supply. The differences in these functional patterns in urban wetlands underscore the dominant influence of anthropogenic N inputs and habitat modification in reshaping the potential of microbial functional traits related to N cycling.

### 4.3. Differences in the Composition and Diversity of Microbial Communities in Urban Wetlands

The distinct responses of microbial communities in aquatic and soil habitats to urbanization highlight the role of environmental buffering in mediating ecological stability [36,49,50]. The heterogeneous microenvironments of soil (e.g., aggregates, redox gradients) buffer external disturbances, thereby sustaining microbial community stability under urbanization. This complexity mitigates the impacts of external disturbances and imposes more consistent selective pressures across urban wetlands [51]. In contrast, aquatic environments are inherently more vulnerable to rapid, stochastic disturbances associated with urbanization, such as elevated nutrient levels, fluctuations in dissolved oxygen, and inputs of anthropogenic contaminants [52]. These fluctuations act as strong environmental filters, favoring taxa with high dispersal ability and metabolic flexibility while suppressing less resilient species [53]. Therefore, aquatic microorganisms, which respond rapidly and distinctly to environmental changes, exhibit greater sensitivity to substantial fluctuations in microbial taxonomic and functional diversity under urbanization, as supported by both taxonomic and functional metrics. For example, Bacteroidetes, known for their ability to degrade labile and complex organic matter (e.g., urban sewage), tend to thrive in response to increased anthropogenic organic inputs [54]. Cyanobacteria abundance in urban wetlands amplifies N_2_O fluxes by enriching N substrates through fixation and decomposition, as well as by driving oxygen fluctuations that promote coupled nitrification-denitrification [46,55]. These shifts in dominant phyla suggest that urban water microbial communities are adapting to prioritize functions critical for survival under elevated resource availability and contamination stress, at the cost of broader functional diversity. Notably, the preserved network connectivity despite compositional shifts indicates a core resilience mechanism through which key biotic interactions persist. Null model analysis further supports this resilience mechanism. Specifically, significant modularity was observed in rural wetlands (Z ≥ 2, *p* < 0.05), indicating that non-random modular aggregation of the network was driven by persistent key biotic interactions and environmental filtering. In contrast, no significant modularity was detected in urban wetlands (Z < 2, *p* > 0.05), suggesting that urban environmental disturbances have weakened the modular aggregation of the network. However, core network connectivity and key biotic interactions may still be preserved in urban wetlands, even in the absence of significant modularity. This reduced modular aggregation, coupled with potentially lower functional redundancy, may render urban wetland ecosystems more vulnerable to abrupt environmental changes. Given the heterogeneity of urban environments and the complexity of wetland ecosystems, we emphasize the importance of considering both aquatic environments and their adjacent soil environments in future research and conservation strategies.

Furthermore, the single-season and limited-site sampling not only restricts the generalizability of our findings across broader spatial and temporal scales but also introduces uncertainties when interpreting the observed microbial functional patterns. Specifically, although this study focuses on gene abundances related to N-cycling processes, it should be acknowledged that gene abundance does not always correspond to actual microbial metabolic activity. This is further exacerbated by the lack of multi-season and multi-site sampling, as temporal and spatial variations in environmental conditions (e.g., Nr loads, hydrological regimes) may alter the relationship between gene abundance and actual metabolic function. Urban wetlands are subject to continuous perturbations, including varying Nr inputs, hydrological alterations, and chemical stressors (e.g., heavy metals, pesticides). However, the single-season and limited-site sampling in this study prevents us from determining whether the observed enrichment of denitrification and nitrification genes reflects a resilient and stable community response or merely a transient adaptation to episodic environmental inputs. Future studies should employ multi-season and multi-site sampling to verify the spatiotemporal stability of microbial functional genes. Additionally, integrating transcriptomics or stable isotope probing techniques would help validate whether the observed genetic shifts correspond to actual changes in N transformation rates, thereby better linking gene abundance with actual microbial activity and N_2_O production rates and improving the generalizability and reliability of research conclusions.

## 5. Conclusions

This study systematically elucidates the distinct responses of microbial communities, N-cycling processes, and N_2_O emissions between urban and rural wetlands, highlighting the profound impacts of urbanization on wetland ecosystems. Notably, urbanization significantly enhances N_2_O emissions from both the soil and water compartments of wetlands. SEM confirmed the direct driving effect of urbanization on emissions from these two interfaces. N_2_O emissions from water are primarily modulated by changes in inorganic nutrients, while those from soil exhibit a positive correlation with the diversity of N-cycling functional genes. Urban wetland microbes show clear compositional adaptations. Aquatic microbial communities are more sensitive to urbanization-induced disturbances, undergoing significant taxonomic shifts while maintaining network stability. This pattern reflects an adaptive strategy for surviving anthropogenic disturbances, yet it is accompanied by a reduction in functional redundancy. In contrast, soil microbial communities maintain relative stability, which can be attributed to the buffering capacity of the soil environment.

Furthermore, urban wetlands exhibit a reorganization of N-cycling functional potentials. The gene abundances of nitrification and denitrification are increased, while those associated with N fixation and organic N metabolism are reduced. Specifically, changes in functional potentials prioritize inorganic N transformation over N retention, and when combined with increased exogenous nutrient inputs, this traps urban wetlands in an “nitrogen loss loop”, further exacerbating high N_2_O fluxes.

Collectively, these findings confirm the central role of microbial communities in mediating the impacts of urbanization on wetland N biogeochemistry, providing a critical scientific basis for wetland ecosystem management. Targeted management strategies, such as reducing nutrient inputs to water bodies and preserving soil microbial diversity, are therefore necessary to mitigate N_2_O emissions while safeguarding ecosystem functionality. Future research should integrate multi-omics approaches (e.g., metatranscriptomics targeting N-cycling genes, metabolomics of nitrogenous compounds) to link the observed compositional differences in N-cycling gene communities between urban and rural wetlands to microbial N transformation activity and explore the long-term stability of urban wetlands’ N-cycling microbial adaptations under anthropogenic disturbances. This will provide a more comprehensive understanding of how urban wetland microbial communities regulate N cycling in response to human-induced environmental changes.

## Figures and Tables

**Table 1:** Null model analysis results of modularity between rural and urban wetlands.

Type	Observed Modularity	Null Model Mean	Z-Score	*p* Value
RS	0.138	0.069 ± 0.028	2.188	0.046
US	0.096	0.079 ± 0.037	0.445	0.253
RW	0.048	0.022 ± 0.012	2.221	0.037
UW	0.146	0.140 ± 0.051	0.128	0.428

Note: Observed modularity reflects the degree of network aggregation and grouping; the null model mean (Q_null) and SD represent the average value and standard deviation of modularity simulated by the null model (random state), respectively. The Z-score is used to quantify the deviation between the observed modularity and the null model mean; a significant difference between the observed modularity and the random state is defined as Z ≥ 2 and *p* < 0.05. RS: rural wetlands soil; US: urban wetlands soil; RW: rural wetlands water; UW: urban wetlands water.

## Data Availability

The sequences reported in this paper have been deposited in the NCBI Sequence Read Archive and is available at https://www.ncbi.nlm.nih.gov/bioproject/PRJNA1021146/ (accessed on 1 January 2026). (Accession Number: SRPPRJNA1021146, metagenomics).

## Supplementary Materials

The following supporting information can be downloaded at https://www.mdpi.com/article/10.3390/microorganisms14030640/s1, Table S1: Geographical coordinates and descriptions of six wetlands among rural and urban wetlands. Table S2: N_2_O flux measurements in wetlands under urbanization. Table S3: Different analysis of N_2_O fluxes about water/soil-atmosphere surface, based on one-way analysis of variance (One-Way ANOVA). Table S4: Water physical and chemical properties at six wetland sampling sites along the rural-urban gradient. Table S5: Soil physical and chemical properties at six wetland sampling sites along the rural-urban gradient. Table S6: Sequencing statistics of sample metagenomic libraries. Table S7: Different analysis of Alpha-diversity about N2O-related microbial diversity in wetlands under urbanization expressed by Shannon, Sobs and Simpson index, based on one-way analysis of variance (One-Way ANOVA). Table S8: Relative abundance of species on Phylum level in wetlands under urbanization. Table S9: Relative contribution of species on Phylum level to functional pathways of N_2_O metabolism in wetlands. Table S10: Correspondence between KEGG Orthology (KO) number and functional genes related to N cycling.

## Abbreviations

The following abbreviations are used in this manuscript:

N	Nitrogen
N_2_O	Nitrous oxide
Nr	Reactive nitrogen
MBC	Microbial biomass carbon
MBN	Microbial biomass nitrogen
SOC	Soil organic carbon
TN	Total nitrogen
TP	Total phosphorus
NH_4_^+^-N	Ammonium nitrogen
NO_3_^−^-N	Nitrate nitrogen
SO_4_^2−^	Sulfate
WT	Water temperature
DO	Dissolved oxygen
ORP	Oxidation-reduction potential
EC	Electrical conductivity
TDS	Total dissolved solids

## References

[1] Murray N.J., Worthington T.A., Bunting P., Duce S., Hagger V., Lovelock C.E., Lucas R., Saunders M.I., Sheaves M., Spalding M. (2022). High-resolution mapping of losses and gains of Earth’s tidal wetlands. Science.

[2] Ravishankara A.R., Danie J.S., Portmann R.W. (2009). Nitrous oxide (N_2_O): The dominant ozone-depleting substance emitted in the 21st century. Science.

[3] Moffett E.R., Simon K.S., Harding J.S. (2015). Urbanisation and earthquake disturbance influence microbial nutrient limitation in streams. Freshwater Biol..

[4] Xian C.F., Zhang X.L., Zhang J.J., Fan Y.P., Zheng H., Salzman J., Ouyang Z.Y. (2019). Recent patterns of anthropogenic reactive nitrogen emissions with urbanization in China: Dynamics, major problems, and potential solutions. Sci. Total Environ..

[5] Glibert P.M. (2017). Eutrophication, harmful algae and biodiversity—Challenging paradigms in a world of complex nutrient changes. Mar. Pollut. Bull..

[6] Lu J.S., Hu T.T., Zhang B.C., Wang L., Yang S.H., Fan J.L., Yan S.C., Zhang F.C. (2021). Nitrogen fertilizer management effects on soil nitrate leaching, grain yield and economic benefit of summer maize in northwest China. Agric. Water Manag..

[7] Dalu T., Wasserman R.J., Magoro M.L., Froneman P.W., Weyl O.L.F. (2019). River nutrient water and sediment measurements inform on nutrient retention, with implications for eutrophication. Sci. Total Environ..

[8] Li X.F., Gao D.Z., Li Y., Zheng Y.L., Dong H.P., Liang X., Liu M., Hou L. (2023). Increased nitrogen loading facilitates nitrous oxide production through fungal and chemodenitriffcation in estuarine and coastal sediments. Environ. Sci. Technol..

[9] Alster C.J., German D.P., Lu Y., Allison S.D. (2013). Microbial enzymatic responses to drought and to nitrogen addition in a southern California grassland. Soil Biol. Biochem..

[10] Bahram M., Espenberg M., Pärn J., Lehtovirta-Morley L., Anslan S., Kasak K., Kõljalg U., Liira J., Maddison M., Moora M. (2022). Structure and function of the soil microbiome underlying N_2_O emissions from global wetlands. Nat. Commun..

[11] Liu L., Barberán A., Gao C., Zhang Z.C., Wang M., Wurzburger N., Wang X., Zhang R., Li J.X., Zhang J. (2022). Impact of urbanization on soil microbial diversity and composition in the megacity of Shanghai. Land Degrad. Dev..

[12] Séneca J., Söllinger A., Herbold C.W., Pjevac P., Prommer J., Verbruggen E., Sigurdsson B.D., Peñuelas J., Janssens I.A., Urich T. (2021). Increased microbial expression of organic nitrogen cycling genes in long-term warmed grassland soils. ISME Commun..

[13] Lu C.Y., Kotze D.J., Setälä H.M. (2020). Soil sealing causes substantial losses in C and N storage in urban soils under cool climate. Sci. Total Environ..

[14] Hu H.W., Zhang L.M., Dai Y., Di H.J., He H.Z. (2013). pH-dependent distribution of soil ammonia oxidizers across a large geographical scale as revealed by high-throughput pyrosequencing. J. Soils Sediments.

[15] Bakken L.R., Bergaust L., Liu B.B., Frostegãrd Ã. (2012). Regulation of denitrification at the cellular level: A clue to the understanding of N_2_O emissions from soils. Philos. Trans. R. Soc. B.

[16] Xue K., Coe J.P., Zhou A., Liu F.F., Li D.J., Wu L.Y., Deng Y., He Z.L., Nostrand J.D., Luo Y.Q. (2016). Warming alters expressions of microbial functional genes important to ecosystem functioning. Front. Mcirobiol..

[17] Sun R.B., Wang F.H., Hu C.S., Liu B.B. (2021). Metagenomics reveals taxon-specific responses of the nitrogen-cycling microbial community to long-term nitrogen fertilization. Soil Biol. Biochem..

[18] Edwards T.M., Puglis H.J., Kent D.B., Durán J.L., Bradshaw L.M., Farag A.M. (2024). Ammonia and aquatic ecosystems-A review of global sources, biogeochemical cycling, and effects on fish. Sci. Total Environ..

[19] Wang H.T., Marshall C.W., Cheng M.Y., Xu H.J., Li H., Yang X.R., Zheng T.L. (2017). Changes in land use driven by urbanization impact nitrogen cycling and the microbial community composition in soils. Sci. Rep..

[20] Dai Z.M., Yu M.J., Chen H.H., Zhao H.C., Huang Y.L., Su W.Q., Xia F., Chang S.X., Brooks P.C., Dahlgren R.A. (2020). Elevated temperature shifts soil N cycling from microbial immobilization to enhanced mineralization, nitrification and denitrification across global terrestrial ecosystems. Glob. Change Biol..

[21] Xu H.J., Li S., Su J.Q., Nie S.A., Gibson V., Li H., Zhu Y.G. (2014). Does urbanization shape bacterial community composition in urban park soils? A case study in 16 representative Chinese cities based on the pyrosequencing method. FEMS Microbiol. Ecol..

[22] Yi X.Y., Ning C., Feng S.L., Gao H.Q., Zhao J.L., Liao J.Y., Peng Y.H., Zhao S.Q., Liu S.G. (2022). Urbanization-induced environmental changes strongly affect wetland soil bacterial community composition and diversity. Environ. Res. Lett..

[23] Lin G., Lin X. (2022). Bait input altered microbial community structure and increased greenhouse gases production in coastal wetland sediment. Water Res..

[24] Li Y., Fan L.H., Zhang W.L., Zhu X.X., Lei M.T., Niu L.H. (2020). How did the bacterial community respond to the level of urbanization along the Yangtze River?. Environ. Sci. Process. Impacts.

[25] Kalembasa S.J., Jenkinson D.S.A. (1973). A comparative study of titrimetric and gravimetric methods for the determination of organic carbon in soil. J. Sci. Food Agric..

[26] Conrad R. (2020). Importance of hydrogenotrophic, aceticlastic and methylotrophic methanogenesis for methane production in terrestrial, aquatic and other anoxic environments: A mini review. Pedosphere.

[27] Villanueva R.A.M., Chen Z.J. (2019). ggplot2: Elegant Graphics for Data Analysis (2nd ed.). Meas.-Interdiscip. Res..

[28] Csardi G., Nepusz T. (2006). The igraph software. Complex Syst..

[29] Harrell F., Dupont C. (2026). Hmisc: Harrell Miscellaneous.

[30] Douglas B., Martin M. (2021). Matrix: Sparse and Dense Matrix Classes and Methods.

[31] Bastian M., Heymann S., Jacomy M. (2009). Gephi: An open source software for exploring and manipulating networks. Proc. Int. AAAI Conf. Web Soc. Media.

[32] Schermelleh-Engel K., Moosbrugger H., Müller H. (2003). Evaluating the fit of structure equation models: Tests of significance and descriptive goodness-of-fit measures. Methods Psychol. Res..

[33] Kang M.X., Peng S., Tian Y.M., Zhang H.Y. (2018). Effects of dissolved oxygen and nutrient loading on phosphorus fluxes at the sediment-water interface in the Hai River Estuary, China. Mar. Pollut. Bull..

[34] Berbel G.B.B., Favara D.I.T., Braga E.S. (2015). Impact of harbour, industry and sewage on the phosphorus geochemistry of a subtropical estuary in Brazil. Mar. Pollut. Bull..

[35] Liao J.Y., Hu A., Zhao Z.W., Liu X.R., Jiang C., Zhang Z.H. (2021). Biochar with large specific surface area recruits N_2_O-reducing microbes and mitigate N_2_O emission. Soil Biol. Biochem..

[36] Zhang L.Y., Delgado-Baquerizo M., Shi Y., Liu X., Yang Y.F., Chu H.Y. (2021). Co-existing water and sediment bacteria are driven by contrasting environmental factors across glacier-fed aquatic systems. Water Res..

[37] Cui P.Y., Fan F.L., Yin C., Song A., Huang P.R., Tang Y.J., Zhu P., Peng C., Li T.Q., Wakelin S.A. (2015). Long-term organic and inorganic fertilization alters temperature sensitivity of potential N_2_O emissions and associated microbes. Soil Biol. Biochem..

[38] Cheng C., Sun T.Y., Li H.J., He Q., Pavlostathis S.G., Zhang J. (2021). New insights in correlating greenhouse gas emissions and microbial carbon and nitrogen transformations in wetland sediments based on genomic and functional analysis. J. Environ. Manag..

[39] Li Z.G., Xia S.J., Zhang R.H., Zhang R.Q., Chen F., Liu Y. (2020). N_2_O emissions and product ratios of nitrification and denitrification are altered by K fertilizer in acidic agricultural soils. Environ. Pollut..

[40] Grilli J., Rogers T., Allesina S. (2016). Modularity and stability in ecological communities. Nat. Commun..

[41] Cloern J.E., Abreu P.C., Carstensen J., Chauvaud L., Elmgren R., Grall J., Greening H., Johansson J.O.R., Kahru M., Sherwood E.T. (2016). Human activities and climatevariability drive fast-paced change across the world’s estuarine-coastal ecosystems. Glob. Change Biol..

[42] Kaye J.P., McCulley R.L., Burke I.C. (2005). Carbon fluxes, nitrogen cycling, and soil microbial communities in adjacent urban, native and agricultural ecosystems. Glob. Change Biol..

[43] Wang H.T., Cheng M.Y., Dsouza M., Weisenhorn P., Zheng T.L., Gilbert J.A. (2018). Soil Bacterial Diversity Is Associated with Human Population Density in Urban Greenspaces. Environ. Sci. Technol..

[44] Wang Y., Lin J.J., Wang F.F., Tian Q., Zheng Y., Chen N.W. (2023). Hydrological connectivity affects nitrogen migration and retention in the land-river continuum. J. Environ. Manag..

[45] Li P., Pan Z., Sun J.Y., Geng Y., Jiang Y.R., Li Y.Z., Zhang Z. (2025). Anthropogenic climate change may reduce global diazotroph diversity. Nat. Commun..

[46] Zhou J., Zheng Y.L., Hou L.J., An Z.R., Chen F.Y., Liu B.L., Wu L., Qi L., Dong H.P., Han P. (2023). Effects of acidification on nitrification and associated nitrous oxide emission in estuarine and coastal waters. Nat. Commun..

[47] Hu Y.J., Xiang D., Veresoglou S.D., Chen F.L., Chen Y.L., Hao Z.P., Zhang X., Chen B.D. (2014). Soil organic carbon and soil structure are driving microbial abundance and community composition across the arid and semi-arid grasslands in northern China. Soil Biol. Biochem..

[48] Wang Y., Wang F.F., Fang Y., Fu Y.Q., Chen N.W. (2024). Storm-induced nitrogen transport via surface runoff, interflow and groundwater in a pomelo agricultural watershed, southeast China. Environ. Pollut..

[49] Li X.Y., Gu A.Z., Zhang Y., Xie B., Li D., Chen J.M. (2019). Sub-lethal concentrations of heavy metals induce antibiotic resistance via mutagenesis. J. Hazard. Mater..

[50] Wu Y.Y., Zhou S.B., Li Y., Niu L.H., Wang L.Q. (2024). Climate and local environment co-mediate the taxonomic and functional diversity of bacteria and archaea in the Qinghai-Tibet Plateau rivers. Sci. Total Environ..

[51] Zheng Q., Hu Y.T., Zhang S.S., Noll L., Böckle T., Dietrich M., Herbold C.W., Eichorst S.A., Woebken D., Richter A. (2019). Soil multifunctionality is affected by the soil environment and by microbial community composition and diversity. Soil Biol. Biochem..

[52] Przeslawski R., Byrne M., Mellin C. (2015). A review and meta-analysis of the effects of multiple abiotic stressors on marine embryos and larvae. Glob. Change Biol..

[53] Emanuele F., De Leopoldo S., Tiberio F., Bruno F., Antonio G., Matilde G., Emilia P., Enrico T., Daniele V., Giulio Z. (2025). Drivers and patterns of community completeness suggest that Tuscan Fagus sylvatica forests can naturally have a low plant diversity. For. Ecosyst..

[54] Fernández-Gómez B., Richter M., Schüler M., Pinhassi J., Acinas S.G., González J.M., Pedrós-Alió C. (2013). Ecology of marine Bacteroidetes: A comparative genomics approach. ISME J..

[55] Wang H.J., Dai M.H., Liu J.W., Kao S.J., Zhang C., Cai W.J., Wang G.Z., Qian W., Zhao M.X., Sun Z.Y. (2016). Eutrophication driven hypoxia in the East China Sea off the Changjiang Estuary. Environ. Sci. Technol..

