# Supplementary material for: Urbanization-Induced Shifts in Microbial Functional Genes of Wetland Nitrogen Cycling Promote Nitrous Oxide (N2O) Emissions
Source: Microorganisms. 2026 Mar 12;14(3):640. doi: 10.3390/microorganisms14030640 (PMC13029612; doi:10.3390/microorganisms14030640)
Supplement: Supplementary file 1 [file microorganisms-14-00640-s001.zip › microorganisms-4129379-supplementary.pdf]

Table S1 Geographical coordinates and descriptions of six wetlands among rural and urban wetlands.

Study sites	Sampling site	Site description	Longitude (◦E)	Latitude (◦N)	ISA
Rural wetland1	NS		110.37	26.09	0.117
Rural wetland 2	DWS	A less anthropogenically influenced reference site.	114.15	28.43	0.124
Rural wetland 3	MS		112.85	24.91	0.138
Urban wetland1	SYH		113.62	28.16	0.361
Urban wetland 2	GT	Within the most urbanized site, urban wetland.	113.03	28.13	0.763
Urban wetland 3	YH		112.91	28.13	0.428

Rural wetland 1,2 and 3: rural wetland sampling site 1, 2, and 3; Urban wetland 1,2 and 3: urban wetland sampling site 1, 2, and 3. ISA: Impervious surface area. The same below.

Table S2 N_2O flux measurements in wetlands under urbanization.

Sample	Site	Field Date	N_2O flux	n	median	min	max
Water	RU1	2023/04/07	0.017 ± 0.014	19	0.010	0.003	0.046
	RU2	2023/04/03	0.012 ± 0.007	15	0.010	0.010	0.030
	RU3	2023/04/13	0.033 ± 0.036	15	0.019	0.000	0.090
	UR1	2023/04/20	0.027 ± 0.031	15	0.013	0010	0.090
	UR2	2023/04/25	0.076 ± 0.069	15	0.038	0016	0.211
	UR3	2023/04/27	0.247 ± 0.088	15	0.299	0.119	0.343
Soil	RU1	2023/04/07	0.330 ± 0.393	16	0.304	-0.701	0.940
	RU2	2023/04/03	0.163 ± 0.146	17	0.206	-0.112	0.342
	RU3	2023/04/13	-0.147 ± 0.192	18	0.189	-0.177	0.620
	UR1	2023/04/20	1.174 ± 0.746	18	1.103	0.186	2.515
	UR2	2023/04/25	1.214 ± 0.635	18	1.181	0.186	2.558
	UR3	2023/04/27	1.381 ± 0.870	20	1.032	0.586	2.959

Values are means ± standard error, $mg \cdot m^{-2} \cdot h^{-1}$

Table S3 Different analysis of N_2O fluxes about water/soil-atmosphere surface, based on one-way analysis of variance (One-Way ANOVA).

Index	Term	Df	F value	*P* value
N_2O fluxes at the water-atmosphere surface	Type	1	17.558	0.000***
	Residuals	102		
N_2O fluxes at the soil-atmosphere surface	Type	1	117.448	0.000***
	Residuals	102		

Type: rural wetlands and urban wetlands. *P < 0.05, **P < 0.01, ***P < 0.001.

Table S4 Water physical and chemical properties at six wetland sampling sites along the rural-urban gradient.

Study sites	RU1	RU2	RU3	UR1	UR2	UR3
WT	10.00 ± 0.19^{e}	9.65 ± 0.01^{e}	13.67 ± 0.71^{d}	17.77 ± 0.06^{c}	18.67 ± 0.07^{b}	19.33 ± 0.13^{a}
pH	7.52 ± 0.46^{a}	8.00 ± 0.75^{a}	7.47 ± 1.01^{a}	8.14 ± 0.22^{a}	8.16 ± 0.12^{a}	7.97 ± 0.11^{a}
TOC	2.82 ± 0.25^{d}	7.94 ± 1.74^{bcd}	6.17 ± 0.60^{cd}	8.94 ± 1.92^{bc}	16.81 ± 1.41^{a}	12.19 ± 2.90^{ab}
DO	2.48 ± 0.03^{d}	2.58 ± 0.02^{d}	5.46 ± 0.31^{c}	15.73 ± 2.39^{a}	16.63 ± 0.68^{a}	10.85 ± 0.40^{b}
ORP	176.87 ± 32.25^{a}	117.60 ± 6.11^{bc}	162.37 ± 51.72^{ab}	85.37 ± 25.48^{c}	116 ± 3.99^{bc}	85.27 ± 4.63^{c}
EC	9.33 ± 6.35^{c}	34.33 ± 2.52^{c}	21.67 ± 3.79^{c}	397.67 ± 3.06^{b}	869 ± 6.08^{a}	866.67 ± 57.74^{a}
TDS	5.00 ± 3.46^{f}	17.00 ± 1.00^{d}	11.00 ± 1.73^{e}	198.67 ± 1.15^{c}	435.33 ± 4.04^{b}	450.33 ± 0.58^{a}
NO_3^--N	0.07 ± 0.05^{c}	0.12 ± 0.02^{c}	0.05 ± 0.01^{c}	0.17 ± 0.1^{c}	6.86 ± 0.22^{a}	3.96 ± 0.04^{b}
NH_4^+-N	0.30 ± 0.03^{c}	0.36 ± 0.06^{bc}	0.36 ± 0.04^{bc}	0.44 ± 0.03^{a}	0.40 ± 0.04^{ab}	0.43 ± 0.02^{ab}
PO_4^{3-}	0.084 ± 0.02^{c}	0.05 ± 0.01^{c}	0.09 ± 0.01^{c}	1.43 ± 0.10^{a}	1.57 ± 0.18^{a}	1.15 ± 0.09^{b}
SO_4^{2-}	1.02 ± 0.11^{d}	0.81 ± 0.02^{d}	0.86 ± 0.01^{d}	17.07 ± 0.06^{a}	36.2 ± 0.36^{b}	41.17 ± 0.42^{c}
K	0.46 ± 0.11^{e}	1.19 ± 0.10^{d}	1.12 ± 0.16^{d}	5.12 ± 0.03^{a}	10.3 ± 0.10^{b}	12.47 ± 0.12^{c}
Cu	0.31 ± 0.03^{c}	0.36 ± 0.13^{c}	0.27 ± 0.07^{c}	0.84 ± 0.06^{b}	1.13 ± 0.28^{a}	1.02 ± 0.06^{ab}

Pb	0.15±0.06[ab]	0.14±0.02[bc]	0.18±0.03[a]	0.06±0.01[d]	0.09±0.01[bcd]	0.09±0.01[cd]
Cd	0.11±0.01[cd]	0.11±0.01[d]	0.13±0.01[abc]	0.13±0.02[bcd]	0.16±0.02[ab]	0.16±0.01[a]
As	ND	ND	ND	3.09±0.16	3.3±0.76	3.2±0.05
Cr	ND	ND	ND	ND	ND	ND

WT: °C, water temperature; TOC: $mg \cdot L^{-1}$, total organic carbon; DO: $mg \cdot L^{-1}$, dissolved oxygen; ORP: mV, oxidation-reduction potential; EC: $\mu S \cdot cm^{-1}$, electrical conductivity; TDS: $mg \cdot L^{-1}$, total dissolved solids; NO_3^-N: $mg \cdot L^{-1}$, nitrate nitrogen; NH_4^+-N: $mg \cdot L^{-1}$, ammonium nitrogen; PO_4^{3-}: $mg \cdot L^{-1}$, phosphate radical; SO_4^{2-}: $mg \cdot L^{-1}$, sulfate radical; K: $mg \cdot L^{-1}$, heavy metal element; Cu: $\mu g \cdot L^{-1}$, heavy metal element; Pb: $\mu g \cdot L^{-1}$; heavy metal element; Cd: $\mu g \cdot L^{-1}$, heavy metal element;

Table S5 Soil physical and chemical properties at six wetland sampling sites along the rural-urban gradient.

Study sites	RU1	RU2	RU3	UR1	UR2	UR3
pH	$4.33{\pm}0.12^{c}$	$5.17{\pm}0.33^{b}$	$4.86{\pm}0.43^{b}$	$7.53{\pm}0.43^{a}$	$7.47{\pm}0.29^{a}$	$7.39{\pm}0.25^{a}$
TOC	$49.19{\pm}2.13^{a}$	$33.58{\pm}2.17^{b}$	$26.58{\pm}2.84^{b}$	$14.30{\pm}2.84^{c}$	$9.06{\pm}1.32^{c}$	$14.40{\pm}1.25^{c}$
TN	$2.20{\pm}2.13^{d}$	$33.58{\pm}2.17^{a}$	$1.01{\pm}2.84^{d}$	$14.30{\pm}2.84^{b}$	$9.06{\pm}1.32^{c}$	$14.40{\pm}1.25^{b}$
TP	$1.52{\pm}0.19^{a}$	$0.82{\pm}0.20^{b}$	$0.20{\pm}0.01^{e}$	$0.38{\pm}0.01^{d}$	$0.73{\pm}0.03^{c}$	$0.34{\pm}0.02^{d}$
NO_3^--N	$24.53{\pm}0.34^{a}$	$17.05{\pm}0.35^{a}$	$23.19{\pm}0.86^{a}$	$8.83{\pm}0.86^{b}$	$8.45{\pm}0.58^{b}$	$8.08{\pm}0.21^{b}$
NH_4^+-N	$33.89{\pm}19.18^{b}$	$33.52{\pm}19.62^{b}$	$4.43{\pm}0.38^{c}$	$5.01{\pm}0.38^{c}$	$8.78{\pm}1.87^{a}$	$5.17{\pm}1.80^{c}$
SO_4^{2-}	$40.75{\pm}63.01^{d}$	$51.36{\pm}43.33^{d}$	$54.90{\pm}10.24^{d}$	$309.75{\pm}10.24^{a}$	$239.04{\pm}36.13^{b}$	$205.86{\pm}33.95^{c}$
MBC	$787.72{\pm}13.49^{a}$	$514.50{\pm}17.19^{b}$	$461.31{\pm}13.19^{b}$	$165.91{\pm}13.19^{c}$	$187.89{\pm}21.29^{c}$	$191.01{\pm}7.19^{c}$
MBN	$96.73{\pm}3.40^{a}$	$65.85{\pm}3.64^{b}$	$56.74{\pm}3.02^{b}$	$21.74{\pm}3.02^{c}$	$25.51{\pm}8.36^{c}$	$22.90{\pm}3.40^{c}$
Cu	$53.05{\pm}6.93^{a}$	$53.85{\pm}4.26^{a}$	$48.46{\pm}10.43^{a}$	$54.96{\pm}10.43^{a}$	$54.79{\pm}15.42^{a}$	$38.72{\pm}7.25^{a}$
K	$8.61{\pm}0.14^{b}$	$28.01{\pm}0.16^{a}$	$6.38{\pm}0.18^{c}$	$6.34{\pm}0.18^{c}$	$6.02{\pm}0.13^{c}$	$6.09{\pm}0.09^{c}$
Cr	$49.91{\pm}7.39^{c}$	$158.82{\pm}6.90^{a}$	$23.59{\pm}1.92^{d}$	$52.87{\pm}1.92^{c}$	$63.71{\pm}2.64^{b}$	$67.41{\pm}2.22^{b}$
As	$12.58{\pm}0.56^{c}$	$18.60{\pm}0.53^{a}$	$8.36{\pm}0.15^{f}$	$9.77{\pm}0.15^{e}$	$10.76{\pm}0.50^{d}$	$14.79{\pm}0.40^{b}$

Cd	141.00±129.12^{bc}	152.91±144.52^{bc}	142.29±34.09^{b}	143.09±34.09^{bc}	376.08±15.15^{a}	100.10±7.21^{c}
Pb	23.16±2.11^{b}	22.25±2.37^{b}	51.80±2.72^{a}	24.65±2.72^{b}	27.81±3.51^{b}	22.47±2.15^{b}

TOC: g·kg^{-1}, total organic carbon; TN: g·kg^{-1}, total nitrogen; TP: g·kg^{-1}, total phosphorus; NO_3^-N: mg·kg^{-1}, nitrate nitrogen; NH_4^+-N: mg·kg^{-1}, ammonium nitrogen; SO_4^{2-}: mg·kg^{-1}, sulfate radical; MBC: mg·kg^{-1}, soil microbial biomass C; MBN: mg·kg^{-1}, soil microbial biomass N; Cu: mg·kg^{-1}, heavy metal element; K: g·kg^{-1}, heavy metal element; Cr: mg·kg^{-1}, heavy metal element; As: mg·kg^{-1}, heavy metal element; Cd: mg·kg^{-1}, heavy metal element; Pb: mg·kg^{-1}, heavy metal element. Values are means ± standard error. ^{abcdef}: different letters indicate significant differences in each physical and chemical property on the rural-urban gradient, based on $P < 0.05$.

Table S6 Sequencing statistics of sample metagenomic libraries.

ID	Raw reads	Clean reads	Fastq size (Gb)	No. Contigs	N50 of contigs	No. ORFs	Avg_length (bp)
US1-1	51490042	50072932	7.77	316,985	480	364,480	379.19
US1-2	51437446	49924256	7.77	255,005	460	291,315	375.21
US1-3	56990508	55667760	8.61	517,648	509	613,755	395.27
US2-1	52778994	51659664	7.97	415,921	484	487,362	384.64
US2-2	56408182	55231668	8.52	496,305	496	587,240	388.93
US2-3	52448418	51282516	7.92	380,489	486	446,049	384.64
US3-1	56933408	55346492	8.6	522,950	465	603,473	374.91
US3-2	53563200	52395652	8.09	379,171	460	433,360	372.9
US3-3	48102986	46979310	7.26	236,014	434	263,899	361.12
RS1-1	49822520	48720874	7.52	450,934	528	519,181	391.82
RS1-2	50451416	49462408	7.62	497,532	518	571,304	389.18
RS1-3	60796478	58823016	9.1	733,985	502	841,575	386.01
RS2-1	62355030	60700434	9.42	578,615	496	665,958	385.15

ID	Raw reads	Clean reads	Fastq size (Gb)	No. Contigs	N50 of contigs	No. ORFs	Avg_length (bp)
RS2-2	53826184	52470076	8.13	372,150	473	421,457	375.63
RS2-3	55791714	54396300	8.42	278,750	462	311,493	369.07
RS3-1	49018474	46928062	7.4	448,392	532	523,327	397.28
RS3-2	50595648	48473520	7.64	401,707	517	462,876	392.85
RS3-3	53043230	51862262	8.01	401,707	517	462,074	391.7
UW1-1	46356514	45394296	7.00	581,081	682	791,934	425.35
UW1-2	52714578	51756366	7.96	624,453	752	886,981	443.44
UW1-3	44946132	44110738	6.79	546,395	761	786,832	443.56
UW2-1	43525942	42871524	6.57	439,074	785	633,034	456.88
UW2-2	43976676	43143704	6.64	448,384	744	632,630	453.81
UW2-3	53788476	52840452	8.12	561,830	689	778,334	436.4
UW3-1	82868626	81042128	1.25	665,789	1030	1,014,348	501.07
UW3-2	46250520	45359768	6.98	482,104	948	722,043	490.45
UW3-3	42284014	41361992	6.38	467,782	933	696,309	488.04

ID	Raw reads	Clean reads	Fastq size (Gb)	No. Contigs	N50 of contigs	No. ORFs	Avg_length (bp)
RW1-1	52513400	50811558	7.93	227,392	486	270,232	391.03
RW1-2	47182202	46078828	7.12	209,680	579	267,564	422.56
RW1-3	47647328	45599332	7.19	329,601	527	444,829	367.54
RW2-1	50049718	49134620	7.56	262,238	533	326,182	403.39
RW2-2	48775934	47114996	7.37	251,907	547	319,263	405.61
RW2-3	51864962	50713014	7.83	245,570	539	313,719	406.06
RW3-1	43148254	39954464	6.52	226,613	1157	356,629	512.6
RW3-2	46036518	44464158	6.95	222,486	609	294,226	418.84
RW3-3	41704598	40261794	6.3	195,561	628	262,587	424.98

Table S7 Different analysis of Alpha-diversity about N_2O-related microbial diversity in wetlands under urbanization expressed by Shannon , Sobs and Simpson index, based on one-way analysis of variance (One-Way ANOVA).

N_2O-associated microbial diversity	Term	Df	Water		Soil	
			F value	*P* value	F value	*P* value
Shannon index	Type	1	12.705	0.003***	52.037	0.000***
	Residuals	16				
Sobs index	Type	1	10.29	0.005 **	84.865	0.000 ***
	Residuals	16				
Simpson index	Type	1	5.237	0.036	60.136	0.000***
	Residuals	16				

Table S8 Relative abundance of species on Phylum level in wetlands under urbanization.

Species on Phylum level	RS	US	*P* value	RW	UW	*P* value
Proteobacteria	0.436±0.022	0.420±0.021	0.627	0.796±0.024	0.460±0.099	**0.030***
Actinobacteria	0.138±0.016	0.222±0.032	0.079	0.044±0.008	0.307±0.092	**0.046***
Acidobacteria	0.222±0.013	0.134±0.010	**0.006****	0.018±0.006	0.002±0.001	0.059
Chloroflexi	0.069±0.003	0.049±0.003	**0.007****	0.005±0.001	0.008±0.006	0.652
Verrucomicrobia	0.027±0.003	0.016±0.002	**0.036***	0.065±0.016	0.053±0.035	0.767
Candidatus_Rokubacteria	0.019±0.001	0.044±0.006	**0.015***	0.002±0.0004	0.0003±0.00004	**0.027***
Bacteroidetes	0.003±0.001	0.009±0.002	0.071	0.022±0.004	0.097±0.055	0.240
unclassified_d__Bacteria	0.027±0.000	0.023±0.001	**0.002****	0.007±0.00002	0.014±0.005	0.251
Gemmatimonadetes_d__Bacteria	0.009±0.001	0.021±0.003	**0.018****	0.0014±0.0005	0.002±0.001	0.518
Planctomycetes	0.010±0.001	0.016±0.004	0.249	0.007±0.002	0.004±0.004	0.632
Nitrospirae	0.016±0.011	0.013±0.005	0.858	0.004±0.001	0.0002±0.00008	0.031*
Cyanobacteria	0.001±0.00014	0.001±0.000	0.724	0.0011±0.0002	0.043±0.032	0.259
Thaumarchaeota	0.004±0.003	0.005±0.002	0.859	0.003±0.001	0.000±0.00001	**0.009****

Candidatus_Eisenbacteria	0.001±0.0003	0.005±0.001	**0.006****	0.0006±0.00015	0.0001±0.00004	**0.039***
unclassified_d__unclassified	0.002±0.00026	0.003±0.00043	0.432	0.0006±0.00016	0.0003±0.00026	0.370
Firmicutes	0.001±0.00007	0.002±0.00021	**0.032***	0.0012±0.00028	0.0005±0.00009	0.091
candidate_division_NC10	0.002±0.001	0.002±0.00023	0.764	0.0003±0.00008	0.000±0.00003	**0.042***
Chlorobi	0.001±0.00042	0.0002±0.00014	0.425	0.0039±0.00294	0.0013±0.00118	0.455
Candidatus_Omnitrophica	0.001±0.00024	0.0014±0.00018	0.065	0.0018±0.00049	0.000±0.000	**0.023***
Candidatus_Dadabacteria	0.001±0.00012	0.0013±0.00023	0.054	0.0008±0.00045	0.0003±0.00021	0.371
Elusimicrobia	0.001±0.00027	0.0005±0.00013	0.280	0.0012±0.00053	0.000±0.000	0.082
Ignavibacteriae	0.001±0.00038	0.001±0.00017	0.628	0.0004±0.00011	0.0001±0.00008	0.071
Armatimonadetes	0.001±0.00008	0.001±0.0001	0.075	0.0003±0.00022	0.0007±0.00069	0.606
Euryarchaeota	0.001±0.00038	0.0006±0.00017	0.196	0.0004±0.00007	0.0002±0.00002	**0.022***
Candidatus_Nomurabacteria	0.0002±0.00018	0.000±0.000	0.374	0.002±0.001	0.000±0.00001	0.137
others	0.006±0.002	0.010±0.002	0.266	0.012±0.003	0.008±0.002	0.274

Table S9 Relative contribution of species on Phylum level to functional pathways of N_2O metabolism in wetlands.

Module ID Pathway	Species on Phylum level	RS	US	*P* value	RW	UW	*P* value
M00615 M00740 Organic nitrogen metabolism	others	0.031±0.007	0.031±0.004	0.729	0.025±0.005	0.015±0.004	0.082
	p__Acidobacteria	0.316±0.014	0.173±0.014	**<.001**	0.015±0.004	0.0001±0.000	**0.005**
	p__Actinobacteria	0.054±0.006	0.142±0.012	**<.001**	0.022±0.013	0.242±0.053	**0.002**
	p__Bacteroidetes	0.006±0.002	0.012±0.003	**0.026**	0.030±0.014	0.075±0.009	0.056
	p__Candidatus_Bathyarchaeota	0.0015±0.0007	0±0	0.062	0±0.00004	0±0	0.347
	p__Candidatus_Dadabacteria	0±0	0.001±0.000	**0.022**	0±0	0±0	-
	p__Candidatus_Rokubacteria	0.022±0.003	0.050±0.006	**0.005**	0.001±0.001	0±0.00004	**0.031**
	p__Chlorobi	0±0	0.001±0.000	**0.036**	0.0003±0.0003	0.002±0.001	0.174
	p__Chloroflexi	0.1198±0.0127	0.064±0.004	**<.001**	0.0041±0.00109	0.018±0.008	0.105
	p__Cyanobacteria	0±0	0.001±0.000	0.071	0.0001±0.0001	0.116±0.046	**0.036**
	p__Gemmatimonadetes_d__Bacteria	0.017±0.002	0.024±0.003	0.091	0.001±0.001	0.002±0.001	0.943
	p__Nitrospirae	0.012±0.004	0.011±0.003	0.665	0.001±0.0004	0±0	**0.024**
	p__Planctomycetes	0.007±0.002	0.016±0.003	0.057	0.004±0.002	0.004±0.002	0.961
	p__Proteobacteria	0.360±0.016	0.428±0.011	**0.002**	0.779±0.031	0.505±0.092	**0.026**
	p__Spirochaetes	0±0	0.001±0.0003	0.118	0.002±0.001	0±0	0.065
	p__Verrucomicrobia	0.0458±0.0037	0.024±0.002	**<.001**	0.109±0.028	0.015±0.004	**0.015**
	p__candidate_division_NC10	0.001±0.001	0.002±0.0003	0.151	0.0001±0.0001	0±0	0.347
	p__unclassified_d__Bacteria	0.008±0.001	0.019±0.001	**<.001**	0.005±0.001	0.007±0.002	0.357
M00529	others	0.013±0.002	0.023±0.001	**0.004**	0.004±0.001	0.001±0.000	**0.013**
	p__Acidobacteria	0.135±0.009	0.096±0.008	**0.011**	0.029±0.006	0.005±0.002	**0.001**

Denitrification	p__Actinobacteria	0.098±0.012	0.208±0.022	**0.003**	0.029±0.006	0.176±0.071	0.078
	p__Armatimonadetes	0.001±0.0005	0.001±0.000	0.744	0±0	0.0001±0.000	0.347
	p__Bacteroidetes	0.003±0.001	0.009±0.002	**0.022**	0.030±0.026	0.129±0.044	0.107
	p__Calditrichaeota	0±0	0.001±0.000	**0.020**	0±0	0±0	-
	p__Candidatus_Aminicenantes	0±0	0.001±0.000	0.051	0±0	0±0	-
	p__Candidatus_Dadabacteria	0.001±0.000	0.002±0.001	0.302	0.0002±0.0001	0±0	0.347
	p__Candidatus_Eisenbacteria	0.001±0.000	0.005±0.001	**0.003**	0.0001±0.0002	0.001±0.001	0.531
	p__Candidatus_Omnitrophica	0.001±0.000	0.003±0.00	**0.008**	0.001±0.0004	0.0001±0.000	0.208
	p__Candidatus_Rokubacteria	0.01±0.003	0.033±0.002	**<.001**	0.001±0.001	0±0	0.101
	p__Chlorobi	0.0021±0.00088	0±0	**0.048**	0.0004±0.0003	0.025±0.014	0.115
	p__Chloroflexi	0.042±0.004	0.047±0.006	0.282	0.005±0.001	0.0405±0.02027	0.107
	p__Cyanobacteria	0.001±0.001	0.0002±0.0001	0.327	0±0	0±0	-
	p__Elusimicrobia	0.003±0.001	0.001±0.0004	0.107	0.0004±0.0003	0±0	0.179
	p__Euryarchaeota	0.001±0.001	0.0005±0.0002	0.684	0.0003±0.0003	0±0	0.347
	p__Firmicutes	0.0003±0.0002	0.003±0.001	**0.022**	0.001±0.0004	0.001±0.0006	0.925
	p__Gemmatimonadetes_d__Bacteria	0.002±0.001	0.029±0.003	**<.001**	0.002±0.002	0±0	0.315
	p__Ignavibacteriae	0±0	0.001±0.000	0.142	0±0	0±0	-
	p__Nitrospirae	0.041±0.015	0.017±0.004	0.142	0.021±0.008	0.001±0.000	**0.023**
	p__Planctomycetes	0.023±0.003	0.019±0.002	0.258	0.008±0.003	0.007±0.004	0.769
	p__Proteobacteria	0.500±0.020	0.416±0.016	**0.005**	0.761±0.043	0.511±0.038	**<.001**
	p__Spirochaetes	0.0001±0.00006	0.0004±0.00013	**0.042**	0±0	0±0	-
	p__Thaumarchaeota	0.006±0.002	0.004±0.001	0.448	0.011±0.003	0±0	**0.013**
	p__Verrucomicrobia	0.040±0.006	0.015±0.003	**<.001**	0.067±0.033	0.084±0.030	0.744
	p__candidate_division_NC10	0.004±0.002	0.001±0.0003	0.255	0.001±0.0004	0±0	**0.023**
	p__candidate_division_Zixibacteria	0.004±0.002	0.0003±0.0001	0.141	0.0002±0.0001	0±0	0.347

M00530 Dissimilatory nitrate reduction	p__unclassified_d__Archaea	0.001±0.0004	0.0001±0.00011	0.119	0±0	0±0	-
	p__unclassified_d__Bacteria	0.068±0.004	0.063±0.003	0.502	0.026±0.004	0.021±0.004	0.462
	others	0.007±0.001	0.021±0.002	**<.001**	0.003±0.001	0.001±0.0004	0.087
	p__Acidobacteria	0.264±0.017	0.125±0.006	**<.001**	0.018±0.005	0.002±0.001	**0.024**
	p__Actinobacteria	0.110±0.013	0.221±0.022	**0.003**	0.020±0.0038	0.213±0.081	**0.048**
	p__Armatimonadetes	0.001±0.0005	0.0001±0.00007	0.474	0±0	0±0	-
	p__Bacteroidetes	0.002±0.001	0.006±0.001	**0.005**	0.019±0.007	0.029±0.009	0.471
	p__Calditrichaeota	0±0	0.001±0.000	0.053	0±0	0±0	-
	p__Candidatus_Bathyarchaeota	0.0001±0.0001	0±0	0.347	0±0	0±0	-
	p__Candidatus_Dadabacteria	0.001±0.000	0.002±0.001	0.146	0.001±0.00009	0±0	0.347
	p__Candidatus_Eisenbacteria	0±0	0.001±0.0003	0.135	0±0	0±0	-
	p__Candidatus_Omnitrophica	0.001±0.00047	0.004±0.0009	**0.029**	0.001±0.0006	0.001±0.0001	0.142
	p__Candidatus_Rokubacteria	0.017±0.003	0.042±0.004	**0.002**	0.001±0.0004	0.0001±0.0001	0.143
	p__Chlorobi	0.002±0.001	0±0	**0.045**	0.001±0.0001	0±0	0.173
	p__Chloroflexi	0.030±0.004	0.031±0.005	0.860	0.003±0.001	0.002±0.001	0.760
	p__Cyanobacteria	0.002±0.001	0.001±0.000	0.234	0.001±0.001	0±0	**0.040**
	p__Elusimicrobia	0.002±0.001	0.002±0.001	0.968	0.001±0.000	0±0	**0.066**
	p__Firmicutes	0.001±0.000	0.002±0.001	0.066	0.0004±0.0002	0.001±0.000	0.462
	p__Gemmatimonadetes_d__Bacteria	0.001±0.00048	0.0189±0.00236	**<.001**	0±0	0±0	-
	p__Nitrospirae	0.035±0.013	0.016±0.003	0.199	0.009±0.002	0.002±0.001	**0.006**
	p__Planctomycetes	0.011±0.002	0.020±0.004	0.054	0.005±0.003	0.002±0.002	0.541
	p__Proteobacteria	0.466±0.022	0.444±0.016	0.400	0.907±0.012	0.739±0.077	0.077
	p__Spirochaetes	0.000±0.00005	0.001±0.0004	**0.015**	0.0002±0.0001	0±0	0.347
	p__Thaumarchaeota	0±0	0.001±0.0004	**0.029**	0±0	0±0	-
	p__Verrucomicrobia	0.016±0.002	0.015±0.003	0.708	0.003±0.001	0.002±0.001	**0.048**

M00804 Complete nitrification, comammox	p__candidate_division_NC10	0.008±0.004	0.002±0.000	0.144	0.001±0.0002	0±0	**0.027**
	p__candidate_division_Zixibacteria	0±0	0.0002±0.0001	0.347	0±0	0±0	-
	p__unclassified_d__Archaea	0±0	0.0002±0.0001	0.238	0±0	0±0	-
	p__unclassified_d__Bacteria	0.025±0.003	0.023±0.002	0.423	0.0074±0.00144	0.008±0.00	0.839
	others	0.124±0.013	0.099±0.008	0.074	0.023±0.008	0.002±0.001	0.052
	p__Acidobacteria	0.182±0.026	0.328±0.035	0.146	0.057±0.016	0.223±0.080	**0.031**
	p__Actinobacteria	0.001±0.0008	0.0002±0.00013	**0.013**	0±0	0±0	-
	p__Armatimonadetes	0.001±0.000	0.001±0.00083	0.491	0.001±0.00054	0.016±0.004	0.052
	p__Bacteroidetes	0±0	0.001±0.0003	0.832	0±0	0±0	-
	p__Calditrichaeota	0.002±0.001	0.004±0.001	**0.036**	0.0002±0.000	0±0	**0.007**
	p__Candidatus_Dadabacteria	0.001±0.001	0.004±0.001	0.143	0.001±0.0006	0.0002±0.000	0.347
	p__Candidatus_Omnitrophica	0.0003±0.00031	0.032±0.005	**0.028**	0.001±0.001	0±0	0.370
	p__Candidatus_Rokubacteria	0.004±0.002	0±0	**<.001**	0.001±0.000	0±0	0.202
	p__Chlorobi	0.018±0.008	0.042±0.007	**0.049**	0.004±0.001	0.0003±0.0001	0.187
	p__Chloroflexi	0.002±0.001	0±0.00005	**0.016**	0±0	0±0	-
	p__Cyanobacteria	0.004±0.002	0.02±0.001	0.252	0.001±0.000	0±0	**0.028**
	p__Euryarchaeota	0.001±0.000	0.004±0.001	0.238	0.001±0.001	0±0	0.178
	p__Firmicutes	0±0	0.0029±0.00149	0.047	0±0	0±0	-
	p__Gemmatimonadetes_d__Bacteria	0.044±0.010	0.044±0.013	0.086	0.026±0.008	0.002±0.001	0.175
	p__Nitrospirae	0.008±0.002	0.022±0.003	0.980	0.005±0.002	0.0001±0.00007	**0.014**
	p__Planctomycetes	0.500±0.031	0.334±0.015	**0.026**	0.822±0.015	0.748±0.081	0.060
	p__Proteobacteria	0.017±0.004	0.008±0.002	**<.001**	0.019±0.004	0.001±0.001	0.415
	p__Thaumarchaeota	0.017±0.003	0.011±0.002	0.097	0.008±0.002	0.0023±0.002	**0.004**
	p__Verrucomicrobia	0.005±0.002	0.003±0.001	0.234	0.002±0.001	0±0	**0.045**
	p__candidate_division_Zixibacteria	0.001±0.0004	0.0003±0.0002	0.405	0±0	0±0	-

	p__unclassified_d__Archaea	0.058±0.0056	0.030±0.003	0.612	0.024±0.005	0.004±0.001	**0.019**
	p__unclassified_d__Bacteria	0.008±0.0025	0.0150±0.002	**0.005**	0.001±0.001	0.01±0.005	**0.007**
	others	0.004±0.001	0±0	**0.012**	0.001±0.000	0.028±0.008	**0.010**
	p__Acidobacteria	0.238±0.016	0.288±0.019	0.094	0.007±0.002	0.001±0.001	0.150
	p__Actinobacteria	0.020±0.005	0.169±0.008	**<.001**	0.011±0.0052	0.0013±0.001	0.122
	p__Bacteroidetes	0±0	0.016±0.002	**<.001**	0.027±0.003	0.052±0.024	0.295
	p__Candidatus_Rokubacteria	0.062±0.006	0.053±0.010	0.713	0.001±0.0004	0±0	0.084
	p__Chloroflexi	0.028±0.009	0.030±0.004	0.704	0.002±0.001	0±0	0.223
	p__Cyanobacteria	0.001±0.0005	0.002±0.001	0.525	0±0.00003	0.398±0.125	**0.013**
M00531 Assimilatory nitrate reduction	p__Elusimicrobia	0.004±0.002	0±0	0.026	0±0	0±0	-
	p__Euryarchaeota	0±0	0±0	-	0±0	0.007±0.002	**0.010**
	p__Firmicutes	0±0	0.001±0.001	0.269	0±0	0±0	-
	p__Gemmatimonadetes_d__Bacteria	0±0	0.002±0.001	**0.020**	0±0	0±0	-
	p__Nitrospirae	0.007±0.004	0.003±0.001	0.289	0.001±0.0002	0±0	**0.037**
	p__Planctomycetes	0.0201±0.0049	0.018±0.002	0.921	0.008±0.002	0±0	**0.013**
	p__Proteobacteria	0.473±0.030	0.361±0.017	**0.020**	0.820±0.046	0.448±0.118	**0.023**
	p__Spirochaetes	0.001±0.001	0±0	0.347	0.0001±0.0001	0±0	0.347
	p__Verrucomicrobia	0.072±0.015	0.029±0.002	**0.004**	0.122±0.033	0.064±0.019	0.216
	p__candidate_division_NC10	0±0	0.0005±0.00049	0.347	0±0	0±0	-
	p__unclassified_d__Bacteria	0.069±0.006	0.027±0.005	**<.001**	0.0021±0.001	0.0003±0.0002	0.126
	others	0.006±0.003	0.003±0.002	0.577	0.014±0.006	0±0	**0.043**
M00175 Nitrogen fixation	p__Actinobacteria	0.007±0.006	0±0	0.301	0.004±0.003	0±0	-
	p__Bacteroidetes	0±0	0±0	-	0±0	0±0	0.260
	p__Chlorobi	0±0	0.0012±0.001	0.347	0.116±0.084	0±0	-
	p__Chloroflexi	0±0	0±0	-	0±0	0±0	0.206

	p__Cyanobacteria	0±0	0±0	-	0.0001±0.0001	0.331±0.165	0.081
	p__Euryarchaeota	0±0	0±0	-	0.007±0.004	0.006±0.006	0.884
	p__Firmicutes	0±0	0.017±0.007	**0.041**	0.013±0.008	0±0	0.145
	p__Nitrospirae	0.069±0.035	0.031±0.011	0.256	0.008±0.004	0±0	0.069
	p__Planctomycetes	0.016±0.011	0.006±0.005	0.381	0.009±0.004	0±0	0.070
	p__Proteobacteria	0.728±0.046	0.868±0.020	**0.017**	0.725±0.080	0.565±0.144	0.406
	p__Spirochaetes	0.008±0.005	0.013±0.006	0.471	0.013±0.006	0±0	0.062
	p__Verrucomicrobia	0.092±0.043	0.026±0.016	0.192	0.074±0.024	0±0	**0.014**
	p__unclassified_d__Archaea	0±0	0±0	-	0.003±0.002	0.021±0.021	0.417
	p__unclassified_d__Bacteria	0.075±0.029	0.037±0.001	0.253	0.013±0.004	0.077±0.042	0.158
	others	0±0	0.079±0.025	**0.015**	0±0	0±0	-
	p__Acidobacteria	0.097±0.044	0.0004±0.00039	**0.012**	0±0	0±0	-
	p__Actinobacteria	0.004±0.003	0±0	0.170	0.040±0.025	0.013±0.011	0.051
	p__Candidatus_Dadabacteria	0.005±0.004	0.084±0.038	0.052	0±0	0±0	-
	p__Candidatus_Rokubacteria	0.008±0.007	0±0	0.347	0±0	0±0	-
M00528	p__Chloroflexi	0.006±0.005	0±0	0.347	0±0	0±0	-
Nitrification	p__Gemmatimonadetes_d__Bacteria	0±0	0.005±0.003	0.171	0±0	0±0	-
	p__Nitrospirae	0.033±0.015	0.340±0.09	**0.003**	0.039±0.01619	0±0	**0.032**
	p__Proteobacteria	0.538±0.045	0.172±0.038	**<.001**	0.752±0.038	0.983±0.011	**<.001**
	p__Thaumarchaeota	0.222±0.063	0.214±0.042	0.994	0.129±0.036	0.004±0.003	**0.005**
	p__unclassified_d__Archaea	0.008±0.007	0±0	0.212	0±0	0±0	-
	p__unclassified_d__Bacteria	0.08±0.039	0.050±0.02	0.579	0.041±0.016	0±0	**0.010**

Table S10 Correspondence between KEGG Orthology (KO) number and functional genes related to N cycling

Pathway	Gene	*P* value		KO	Definition
		Water	Soil		
Organic N metabolism	glnA	0.077	**<0.001**	K01915	glutamine synthetase [EC:6.3.1.2]
	gltB	0.063	**<0.001**	K00265	glutamate synthase (NADPH) large chain [EC:1.4.1.13]
	gltD	0.004	**<0.001**	K00266	glutamate synthase (NADPH) small chain [EC:1.4.1.13]
	GLT1	0.113	0.340	K00264	glutamate synthase (NADH) [EC:1.4.1.14]
	GLU	0.489	0.605	K00284	glutamate synthase (ferredoxin) [EC:1.4.7.1]
	GDH2	0.004	**0.001**	K15371	glutamate dehydrogenase [EC:1.4.1.2]
	gudB	**0.011**	0.489	K00260	glutamate dehydrogenase [EC:1.4.1.2]
	GLUD1_2	**0.004**	**0.000**	K00261	glutamate dehydrogenase (NAD(P)+) [EC:1.4.1.3]
N fixation	nifD	0.002	0.402	K02586	nitrogenase molybdenum-iron protein alpha chain [EC:1.18.6.1]
	nifH	0.001	0.354	K02588	nitrogenase iron protein NifH
	nifK	<0.001	0.31	K02591	nitrogenase molybdenum-iron protein beta chain [EC:1.18.6.1]

	vnfD	0.317	<0.001	K22896	vnfD; vanadium-dependent nitrogenase alpha chain [EC:1.18.6.2]
	vnfH	0.317	0.317	K22899	vanadium nitrogenase iron protein
	anfG	0.317	1.0	K00531	vanadium nitrogenase iron protein
Dissimilatory nitrate reduction	nirB	0.001	<0.001	K00362	nitrite reductase (NADH) large subunit [EC:1.7.1.15]
	nirD	0.001	0.508	K00363	nitrite reductase (NADH) small subunit [EC:1.7.1.15]
	nrfA	0.005	<0.001	K03385	nitrite reductase (cytochrome c-552) [EC:1.7.2.2]
	nrfH	0.172	<0.001	K15876	cytochrome c nitrite reductase small subunit
Assimilatory nitrate reduction	narB	0.2	<0.001	K00367	ferredoxin-nitrate reductase [EC:1.7.7.2]
	nasB	1	0.11	K00360	assimilatory nitrate reductase electron transfer subunit [EC:1.7.99.-]
	nasC	<0.001	<0.001	K00372	assimilatory nitrate reductase catalytic subunit [EC:1.7.99.-]
	nirA	0.009	0.031	K00366	ferredoxin-nitrite reductase [EC:1.7.7.1]
Nitrification	pmo-amoA	<0.001	0.627	K10944	methane/ammonia monooxygenase subunit A [EC:1.14.18.3 1.14.99.39]
	pmo-amoB	0.005	0.757	K10945	methane/ammonia monooxygenase subunit B

	pmo-amoC	0.001	0.354	K10946	methane/ammonia monooxygenase subunit C
	hao	0.105	0.536	K10535	hydroxylamine dehydrogenase [EC:1.7.2.6]
Denitrification	narG	0.038	<0.001	K00370	nitrate reductase / nitrite oxidoreductase, alpha subunit [EC:1.7.5.1 1.7.99.-]
	narH	0.757	<0.001	K00371	nitrate reductase / nitrite oxidoreductase, beta subunit [EC:1.7.5.1 1.7.99.-]
	napA	0.085	<0.001	K02567	nitrate reductase (cytochrome) [EC:1.9.6.1]
	napB	0.566	<0.001	K02568	nitrate reductase (cytochrome), electron transfer subunit
	narI	0.145	<0.001	K00374	nitrate reductase gamma subunit [EC:1.7.5.1 1.7.99.-]
	nirK	**0.005**	<0.001	K00368	nitrite reductase (NO-forming) [EC:1.7.2.1]
	nirS	0.691	<0.001	K15864	nitrite reductase (NO-forming) / hydroxylamine reductase [EC:1.7.2.1 1.7.99.1]
	norB	0.354	<0.001	K04561	nitric oxide reductase subunit B [EC:1.7.2.5]
	norC	0.354	0.354	K02305	nitric oxide reductase subunit C
	nosZ	0.895	<0.001	K04561	nitric oxide reductase subunit B [EC:1.7.2.5] | (GenBank) nitric oxide reductase, NorZ apoprotein
